# Synthesis and Characterization of Novel Wholly Aromatic Copolyesters Based on 4′-Hydroxybiphenyl-3-Carboxylic and 3-Hydroxybenzoic Acids

**DOI:** 10.3390/polym15092133

**Published:** 2023-04-29

**Authors:** Pavel A. Mikhailov, Kirill V. Zuev, Valery G. Kulichikhin

**Affiliations:** A. V. Topchiev Institute of Petrochemical Synthesis, Russian Academy of Sciences (TIPS RAS), 29 Leninsky Prospekt, 119991 Moscow, Russia; klch@ips.ac.ru

**Keywords:** aromatic polyester, polycondensation, polymer analysis, amorphous polymer, NMR, rheology, DSC, TGA

## Abstract

A series of new wholly aromatic (co)polyesters based on *m*-substituted bifunctional comonomers—4′-hydroxybiphenyl-3-carboxylic (3HBCA) and 3-hydroxybenzoic (3HBA) acids with molar ratios of 3HBCA:3HBA from 0:100 to 60:40, respectively—was synthesized. NMR and FTIR spectroscopy methods proved the full compliance of the copolymer composition with the target ratio of comonomers, as well as high compositional homogeneity (absence of block sequences). The resulting copolyesters have a sufficiently high molecular weight and their intrinsic viscosity values are in the range of 0.6–0.8 dL/g. Thermal analysis showed that all 3HBCA-3HBA copolyesters are amorphous, and with an increase in the content of biphenyl units (3HBCA), the glass transition temperature increases significantly (up to 190 °C). The onset of the intense thermal decomposition of the synthesized polyesters occurs above 450 °C. Thus, this indicates a sufficiently high thermal stability of these polyesters. Rheological measurements have shown that melts of copolyesters with a high content of 3HBCA units exhibit anisotropic properties. At the same time, the method of polarization optical microscopy did not confirm the transition to the liquid crystal state for these polyesters. These results confirm that it is possible to obtain high-performance polyesters based on 3HBCA, but not a mesogenic comonomer. Thus, 3HBCA is a promising comonomer for the synthesis of new thermotropic copolyesters with controlled anisotropic properties.

## 1. Introduction

Thermotropic main-chain polyesters (TMCPs) obtained from aromatic monomers have gained both academic and industrial interest for several decades and a lot of TMCPs have been previously reported [1,2,3]. The most famous TMCPs are copolyesters of 4,4′-biphenylene terephthalate/isophthalate (BPT/I) and 4-hydroxybenzoic acid (HBA) invented by Economy and coworkers in the 1970s [4,5] and “Vectra” copolyesters of HBA and 6-hydroxy-2-naphtoic acid (HNA) invented by Calundann [6]. Nowadays, compounds based on BPT/I–HBA and HBA–HNA are commercially available from several companies. TMCPs possess a number of unique properties, such as good processability, high modulus, excellent heat resistance, low thermal expansion coefficient, high thermal, radiation, and chemical resistance [7]. Usually, TMCPs represent copolymers, because the homopolymers composed from the rigid mesogenic unit, e.g., *p*-phenylene terephthalate, *p*-hydroxybenzoate, and 2,6-hydroxynaphthoate, are unmeltable below its decomposition temperature [1,8,9]. For example, Vectra A-950 is a copolymer composed of two mesogenic units: HBA (73%) and HNA (27%). Frequently, additional non-mesogenic so-called “kinked” units are inserted into the TMCP backbone. The incorporation of comonomers with a 120° angle bond, such as isophthalic acid, resorcinol, and 3-hydroxybenzoic acid (3HBA), is a widely used approach in the melting or softening points of main-chain TMCP reduction [7]. Recently, another kinked comonomer with a bond angle differing in 120° were proposed. 2,5-Furandicarboxylic acid, a bio-based monomer with a bond angle of 124° [10], was successfully used for the preparation of thermotropic polyesters [11,12]. A series of works was published describing the syntheses of copolyesters based on kinked 3,4-biphenyldicarboxylic acid (3,4BDCA) and aliphatic and aromatic diols [13,14,15,16]. Depending on their composition, polymers with various structures were obtained: amorphous and semicrystalline in the solid state forming isotropic and anisotropic (nematic and smectic) melts.

Incorporation of 3,4-biphenylene units into a polyethylene terephthalate backbone tends to increase their glass transition temperature and tensile strength and decrease their oxygen permeability [13]. It is remarkable that a high content of 3,4-biphenyldicarboxylic acid in copolyesters disrupt the crystalline order preserving liquid crystalline state formation and results in an “anisotropic glass state” [15]. Homopolymers obtained by polycondensation of hydroquinone derivatives and 3,4BDCA are very promising TMCPs with a high glass transition temperature [16]. Inspired by these papers, we started an investigation of copolyesters based on 4′-hydroxybiphenyl-3-carboxylic acid (3HBCA), as a structure-similar to 3,4-biphenyldicarboxylic acid, but with a lower melting point. On the other hand, 3HBCA is an isomer of 4′-hydroxybiphenyl-4-carboxilic acid (4HBCA) and can be isolated as a by-product of the synthesis of 4HBCA [17]. A homopolymer and copolyesters of 4HBCA were previously described in a number of papers [17,18,19,20,21]. According to those works, 4HBCA is one of the promising mesogenic comonomers; however, to the best of our knowledge, homopolymers and copolymers of 3HBCA have never been obtained, and the question “Is 3HBCA a mesogene or not?” remains open.

The most convenient route of fully aromatic copolyester synthesis is the melt polycondensation of acetylated hydroxy acids and/or a mixture of acetylated biphenols and dicarboxylic acids. In the earliest work on the subject [16], copolyesters of 3HBA and 4HBA were successfully prepared by the polycondensation of a mixture of corresponding acetoxy derivatives (3ABA and 4ABA). In the case of BPT/I–HBA copolyesters, a mixture of terephthalic/isophthalic acid, 4,4′-diacetoxybiphenyl, and 4ABA was used [4,5]. For industrial application, the one-pot process is more preferable, while prior to polycondensation, a mixture of biphenols, diacids, hydroxy acids, and an excess of acetic anhydride was refluxed involving the in situ generation of corresponding acetoxy derivatives [4,16].

Moreover, in the case of the melt polycondensation of dicarboxylic acids and diacetates of diol mixtures, the process is heterogeneous, because the former component possesses a higher melting temperature. This can result in the formation of block sequences in the polymer backbone. The block sequences in main-chain thermotropic copolyesters can worsen their mechanical properties [22]. For example, the presence of the blocky 4HBA sequences in BPT/I–HBA copolyesters was described in [4]. Poly[(*m*-phenylene terephthalate)-co-4-oxybenzoate)] synthesized from terephthalic acid, 4HBA, and resorcinol diacetate also demonstrated blockiness [23]. In contrast, copolyesters of HBA and HNA, prepared by the homogeneous melt polycondensation of their acetoxy derivatives, are completely random, which was confirmed by the HR-NMR method [24]. Thus, the polycondensation of acetoxy acid instead of diacetyl diol and dicarboxylic acid looks more preferable.

In the present work, we synthesized copolyesters of 3HBCA and 3HBA, containing up to 60 mol.% of 3HBCA units by the melt polycondensation of corresponding acetyl derivatives. Due to the solubility of copolyesters in chloroform or a chloroform/trifluoroacetic acid (TFA) mixture, gel permeation chromatography (GPC) and extensive HR-NMR analysis, including end group and dyad sequence distribution, were available for these copolyesters. In addition, the rheological behavior of melts as well as their thermal and optical properties were studied.

## 2. Materials and Methods

### 2.1. Materials

3-Hydroxybenzoic acid (3HBA, >98% purity) was produced by Reachem (Moscow, Russia). DMSO-*d*_6_ and chloroform-*d* (>99.8% purity) were purchased from Cambridge Isotope Laboratories (Tewksbury, MA, USA). Trifluoroacetic acid (TFA, >98% purity) was offered by Fluka (Switzerland, Basel). All other solvents (analytical-grade) were purchased from Ekos-1 LLC (Russia, Moscow).

3-Acetoxybenzoic acid (3ABA) was prepared by refluxing (2 h) 3-hydroxybenzoic acid in toluene with a 20% molar excess of acetic anhydride according to [16] and then recrystallizing from water. ^1^H NMR (300 MHz, CDCl_3_) *δ*, ppm: 11.68 (s, 1H), 8.00 (d, *J* = 7.8 Hz, 1H), 7.84 (d, *J* = 2.4 Hz, 1H), 7.50 (t, *J* = 7.9 Hz, 1H), 7.36 (dd, *J* = 8.0, 2.5 Hz, 1H), 2.34 (s, 3H).

4′-Acetoxybiphenyl-3-carboxylic acid (3ABCA) was provided by Yaroslavl State Technical University (Russia). ^1^H NMR (400 MHz, CDCl_3_) δ, ppm: 8.30 (t, *J* = 1.8 Hz, 1H), 8.10 (dt, *J* = 7.9, 1.4 Hz, 1H), 7.86 (dt, *J* = 8.0, 1.4 Hz, 1H), 7.68–7.62 (m, 2H), 7.58 (t, *J* = 7.8 Hz, 1H), 7.23–7.17 (m, 2H), 2.45 (s, 3H).

### 2.2. Polymer Synthesis

A small amount of comonomer mixtures (about 1 g) with 3HBCA:3HBA molar ratios of 100:0, 95:5, 90:10, 80:10, and 70:30 were polymerized in a 50 mL 2-neck round-bottom flask connected to an argon vacuum line. The flask was heated to 360 °C under the slow flow of argon or the vacuum.

The melt polycondensation on a 10–15 g scale was performed using the following procedure. A mixture of 3HBCA and 3HBA was loaded into a 100 mL 3-neck round bottom flask equipped with an overhead mechanical stirrer, inert gas inlet, and vacuum outlet. The flask was evacuated and filled with nitrogen three times, then immersed into a metal bath preheated to 270 °C under the slow flow of nitrogen. A small piece of Mg (1–3 mg) was added into the flask. After 30 min of stirring, a vacuum (<1 mm Torr) was applied. After 60–90 min, a mixture became viscous and began to climb along the stirrer. The temperature of the metal bath was raised to 350–360 °C and stirring continued until the reaction medium became too viscous to be agitated. The flask was filled with nitrogen and the product was removed under the stream of nitrogen and cooled. This method was used to obtain copolymers with initial molar concentrations of 3ABCA from 20 to 60% (named BP20–BP60, respectively). The homopolymer poly(3HBA) was prepared in a similar way.

### 2.3. Polymer Characterization

The measurement of intrinsic viscosity (*IV*, [*η*]) for polymers was carried out at 25 ± 0.1 °C in a mixture of 1,2-dichorobenzene/phenol (1:1 *w*/*w*) using an Ubbelohde viscometer in accordance with ISO 1628-5-1998.

^1^H NMR spectra were recorded on a MSL-300 spectrometer (Bruker, Billerica, MA, USA) with an operating frequency of 300 MHz and sampling time of 1.8 s with 20 accumulations and on an AVANCE III HD spectrometer (Bruker, Germany) with an operating frequency of 400 MHz and sampling time of 10 s with 4–64 accumulations. ^13^C, 2D COSY HSQC NMR spectra were recorded on an AVANCE III HD spectrometer. In the case of the ^13^C spectra, the sampling time was 2 s and 15,000 accumulations were carried out. CDCl_3_ or the mixture CDCl_3_:CF_3_COOH (5:1 *v*/*v* or 5:2 *v*/*v*) were used for monomers and polymers. ^1^H NMR analysis of hydrolyzed polymers was conducted after hydrolyzation by a mixture of CD_3_OD/D_2_O/NaOD [22]. The mixture was prepared by dissolving 280 mg of sodium in 2.0 mL of CD_3_OD with the subsequent addition of 0.9 mL of D_2_O. The concentrations were 40–50 mg/mL.

FTIR spectra were recorded in the reflection mode of a Hyperion 2000 IR Microscope coupled with an IFS-66 v/s IR-Fourier spectrometer (Bruker, Billerica, MA, USA): crystal Ge, resolution 2 cm^−1^, wavelength range 4000–600 cm^−1^.

GPC analysis of polymers was performed on a Waters system with a differential refractometer (Chromatopack Microgel-5; eluent–chloroform; flow rate—1 mL/min). The molecular weights and polydispersity were calculated by a standard procedure relative to monodispersed polystyrene standards.

DSC thermograms of copolyesters were measured and analyzed using an MDSC 2920 instrument (TA Instruments, New Castle, DE, USA) in the following mode: two identical cycles of heating to 350 °C and cooling to 20 °C at 20 K/min in an inert atmosphere (argon, gas flow rate 50 cm^3^/min).

Thermogravimetric analysis was performed on a TGA/DSC3+ thermal analysis instrument (Mettler Toledo, Columbus, OH, USA) in the temperature range from 30 to 1000 °C at a heating rate of 10 °C/min. The inert gas flow rate (argon) was 100 cm^3^/min.

The optical properties of the thin layers of copolyester melts were studied using a 6 PO polarizing optical microscope (Biomed, Moscow, Russia) equipped with a FP900 heating table (Mettler, Columbus, OH, USA) and an E3ISPM5000 photo/video camera (ToupTek Photonics Co., Zhejiang, China), in the following mode: heating to 375 °C and cooling to 25 °C (10–20 K/min).

The rheological characteristics of the copolyester melts were measured at 320 °C using a Haake RheoStress 600 rotational rheometer (Thermo Fisher Scientific, Karlsruhe, Germany) in the stationary shear (shear rate range 0.01–100 s^−1^) and oscillation (amplitude range 0.1–1000 Pa, frequency range 1–100 Hz) modes using a disposable plate–plate operating unit (diameter 20 mm, gap 1 mm). Copolyester samples were dried at 160 °C for 24 h prior to measurements.

## 3. Results and Discussion

### 3.1. Polymer Synthesis and Characterization

**Figure 1:**
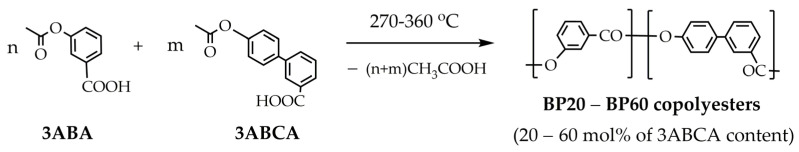
Synthesis of copolyesters by melt polycondensation of 3ABA with 3ABCA.

In our research, we employed the polycondensation of acetoxy derivatives of 3HBA and 3HBCA for the synthesis of copolyesters with different initial molar concentrations of 3HBCA. As mentioned above, 3HBCA is a structure similar to 3,4-biphenyldicarboxylic acid (3,4BDCA), but in contrast to 3,4BDCA, 3HBCA (and the corresponding acetyl derivative) represents an AB-type monomer; thus, it does not require the accompaniment of diol for polymer synthesis. The melt polycondensation of 3ABCA and other aromatic acetoxy acids proceeds in a single phase, because the acetoxy acids possess a low melting temperature. The scheme of the melt acidolysis polycondensation of 3ABCA and 3ABA is presented in Figure 1.

To estimate the range of compositions that allowed for melt-processible copolyesters to be obtained, about 1 g of the 3ABCA:3ABA mixtures with variable molar ratios (100:0, 95:5, 90:10, 80:20, and 70:30) were polymerized in a 50 mL flask under the slow stream of argon. The mixture, containing 80–100% of 3ABCA, was solidified by this small-scale polymerization. Further increases in temperature up to 360 °C did not result in softening or melting. When the amount of 3HBCA decreased by up to 70%, softening was observed at about 350 °C. However, the melt was extremely viscous even at 350–360 °C, making it difficult to obtain a polymer with a sufficient molecular weight. Therefore, the amount of 3HBA was further increased and a series of polyesters with 40–80% of 3HBA was prepared (Table 1). In addition, poly(3HBA) was prepared by the homopolycondensation of 3ABA. Polyesters containing 60 mol.% and more of 3HBA (BP40–BP20) demonstrated solubility in chloroform, unlike BP50 and BP60 which are soluble only in a chloroform/trifluoroacetic acid (TFA) mixture. All copolyesters BP60–BP20 and homopolymer poly(3HBA) were soluble in a mixture 1,2-dicholorobenzene/phenol (1:1 *w*/*w*) and intrinsic viscosities (*IV*) were measured using a Ubbelohde viscometer (values of [*η*] are shown in Table 1).

**Figure 2:**
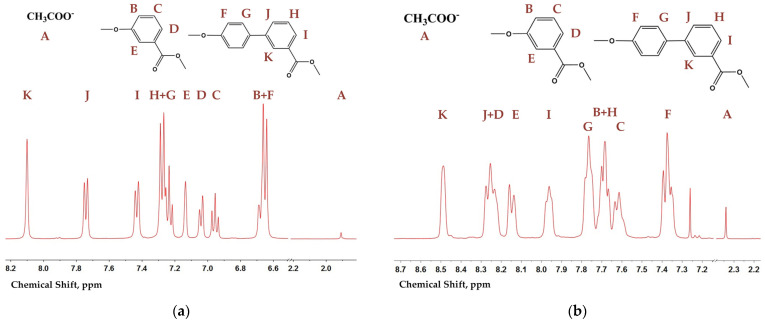
Examples of ^1^H NMR spectra of BP50 (**a**) after hydrolysis by CD_3_OD/D_2_O/NaOD and (**b**) in a CDCl_3_:CF_3_COOH mixture.

The molecular weight and composition of hydrolyzed BP60, BP40, and poly(3HBA) were determined by acetyl end group analysis performed by ^1^H NMR after hydrolysis in the mixture of CD_3_OD/D_2_O/NaOD as previously described [22]. An example of the NMR spectra for copolyester BP50 after hydrolysis with signal assignment is shown in Figure 2a. Signal B of 3HBA and signal F of 3HBCA overlapped, but others did not. Signal A was attributed to sodium acetate released from the acetyl end group after hydrolysis. Such spectra of copolyesters in CD_3_OD/D_2_O/NaOD allowed us to calculate molecular weight (*M*_n_) and composition (Table 1), but did not give information about sequence distribution and randomness.

From analyzing the structure of the macromolecular backbone, ^1^H NMR spectra of copolyesters and poly(3HBA) in CDCl_3_:CF_3_COOH were obtained (example shown in Figure 2b). The spectra of the copolyesters obtained in CDCl_3_:CF_3_COOH were broader in contrast to the sharp spectra obtained in CD_3_OD/D_2_O/NaOD, and they could not give information about the dyads and triads of monomeric units. Signal A of the acetic end group also presented in the spectra allowed for the evaluation of the composition and *M*_n_ (Table 1). Calculation of the composition by ^1^H NMR spectroscopy revealed good correlation between the targeted and obtained monomeric molar ratios with a tendency of lowering of their 3HBA content evidently due to their lower 3ABA thermal stability and higher volatility under polycondensation conditions compared with 3ABCA. The differences between the results of NMR spectroscopy of polymers in the CD_3_OD/D_2_O/NaOD and CDCl_3_:CF_3_COOH solutions were insignificant.

Molecular weights of the BP40, BP30, and BP20 samples and poly(3HBA) were measured by the GPC method using a polystyrene standard. The *M*_n_ values obtained by GPC (Table 1) were higher up to about 1.5 times than those obtained by ^1^H NMR spectroscopy. In some cases, GPC may overestimate molar weight values while NMR spectroscopy is more accurate method. Furthermore, the polydispersity index (*PDI*) of the polymers increased with an increase in 3HBA content from 2.6 (BP40) to 6.3 (poly(3HBA)). On the other hand, end group analysis for copolyester prepared from acetoxy acid is suitable only for rough estimation because of side reactions [25].

**Figure 3:**
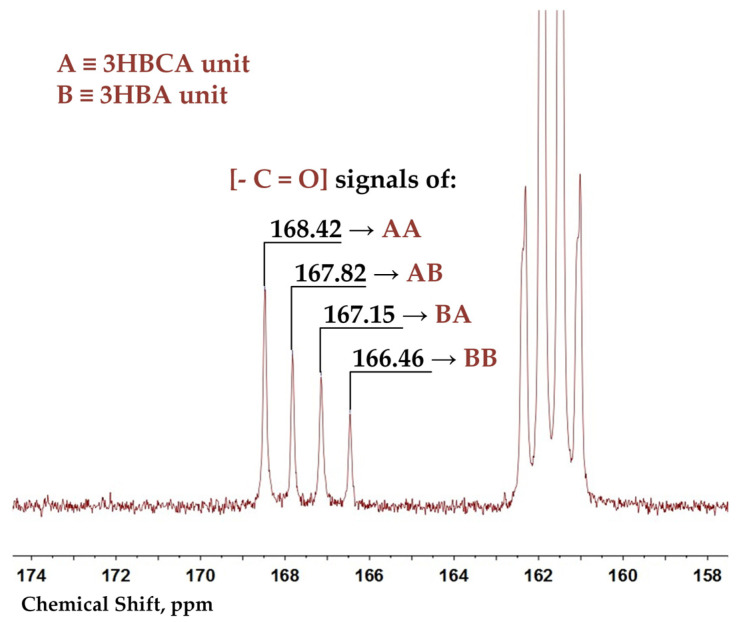
Carbonyl range of a ^13^C NMR spectrum for a BP60 copolyester in a CDCl_3_/CF_3_COOH mixture.

In order to distinguish triads and dyads of monomeric units in a copolyester backbone, the ^13^C spectra of BP40 and BP60 were recorded. Additionally, 2D COSY HSQC NMR spectra of 3-ABCA and copolyesters were recorded to make assignments (see Supplementary Materials, Figures S1–S6). The assignments were made by the comparison of ^1^H, ^13^C, and 2D spectra of copolyesters and monomers (Supplementary Materials, Table S1). The signals 168.31 (166.48), 167.65 (167.82), 166.99 (167.15), and 166.30 (166.46) in BP50 (BP60) spectra were assigned to the carbonyl groups of 3HBCA-3HBCA, 3HBCA-3HBA, 3HBA-3HBCA, and 3HBA-3HBA dyads, respectively (see enlarged spectrum region in Figure 3).

The degree of randomness can be expressed by sequence order parameters according to Equations (1) and (2) [24]:

*Ψ*_A_ = [AB (AA + AB) − AA (BA + BB)]/[AB (AA + AB) + AA (BA + BB)], (1)

*Ψ*_B_ = [BA (BA + BB) − BB (AA + AB)]/[BA (BA + BB) + BB (AA + AB)], (2)

where AA, AB, BA, and BB are the relative intensities of signals in the ^13^C spectra corresponding to dyads 3HBCA-3HBCA, 3HBCA-3HBA, 3HBA-3HBCA, and 3HBA-3HBA, respectively.

In the case of infinite polycondensation degrees, the number of AB and BA sequences is equal, so *Ψ*_A_ = *Ψ*_B_. The value of *Ψ* = 1 is valid for alternating copolyesters (ABABAB…), *Ψ* = −1 for block structures (AAA…BBB…), and *Ψ* = 0 for random copolyesters. Values of *Ψ*_A_ and *Ψ*_B_ < 0.04 for BP50 and BP60 (Table 2) clearly demonstrate that the structure is completely random. These data correlate with the results for HBA-HNA copolyesters (*Ψ* ϵ [−0.02; 0.04]) [24] and contrast to those for the 4-oxybenzoate-1,3-phenylene terephthalate copolyester (*Ψ* ϵ [−0.1; −0.02]) [23]. The consistent trend of blockiness (*Ψ* < 0) was observed for copolyesters obtained by the heteropolycondensation of the diol diacetate and dicarboxylic acid in contrast to copolyesters obtained from acetoxy acids. Remarkably, the ^1^H and ^13^C spectra gave the same values for the compositions of BP50 and BP60 (51–52% 3HBCA for BP50 and 61–62% 3HBCA for BP60).

**Figure 4:**
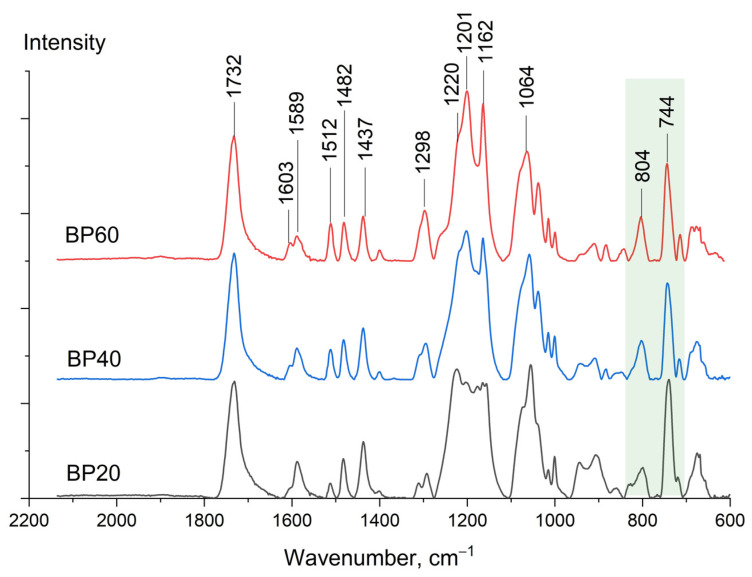
FTIR spectra of BP60, BP40, and BP20 copolyesters.

Fourier-transform infrared spectra (FTIR) were recorded for copolyesters BP60, BP40, and BP20 (Figure 4). A band of the carbonyl group (*ν*_C=O_) corresponded to 1732 cm^−1^ and a series of benzoic ring vibrations (*ν*_CCH_) was located in the range of 1600–1400 cm^−1^. Attention should be paid to the ratio I_744_/I_804_ reflecting the relative ratio of 1,3- and 1,4-substituted derivatives. Table 3 shows the ratio I_744_/I_804_ in the spectra normalized to the carbonyl group, as well as copolyester compositions estimated via the FTIR data. The accuracy of this estimation is not high (especially in comparison with NMR results) but it can be used to estimate composition when more sensitive methods (e.g., NMR) are unavailable.

### 3.2. Thermal Characteristics

**Figure 5:**
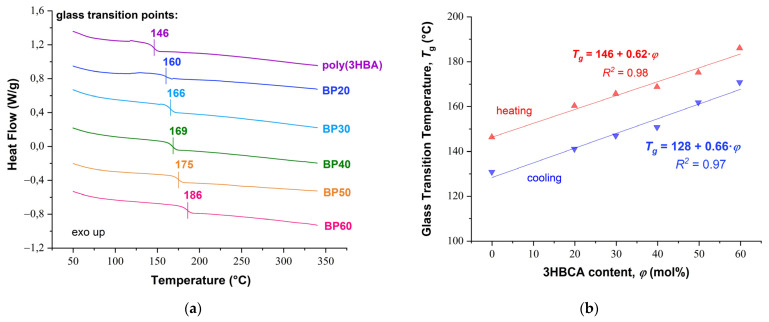
(**a**) DSC thermograms for copolyesters BP20–BP60 and homopolymer poly(3HBA) in the second heating cycle at a rate of 20 K/min in an inert atmosphere. (**b**) Dependences of glass transition temperatures on 3HBCA content obtained during heating and cooling cycles.

DSC thermograms for copolyesters BP20–BP60 and homopolymer poly(3HBA) are shown in Figure 5a. The thermograms for all polymers do not demonstrate any endo- or exothermic effects that would indicate either an amorphous or mesophase structure, i.e., the absence of phase transitions in the range of 50–350 °C. At the same time, an increase in the 3HBCA content in (co)polymers resulted in significant increases in the glass transition temperature (*T*_g_): from 146 °C for poly(3HBA) to 186 °C for BP60. The dependence of the glass point on 3HBCA content was linear for these (co)polyesters (Figure 5b).

Increases in the glass transition temperatures of polyesters with the introduction of biphenyl units were repeatedly mentioned earlier. The observed effect was less pronounced in the case of semi-aromatic copolyesters [13,20,21]. For example, fully aromatic copolymers of 4-hydroxybenzoic acid and 4,4′-biphenol terephthalate demonstrated glass transition points up to 180 °C [4].

**Figure 6:**
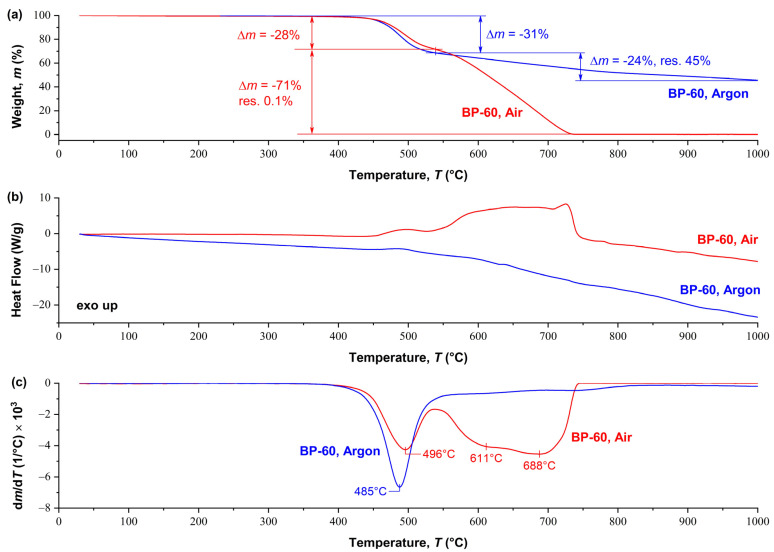
Results of (**a**) TGA, (**b**) DSC, and (**c**) DTG analysis for BP-60 copolyester obtained in an air and inert (argon) atmosphere.

The results of synchronous TGA/DSC analysis for the BP60 copolymer in air and an inert atmosphere are shown in Figure 6. The thermal stability of this copolyester in air and in argon is approximately the same of up to 550 °C, with mass loss at 550 °C by ~30%, 5% mass loss temperature (*T*_Δ5%_) > 450 °C. Above 550 °C, another one-step weight loss occurs, but its magnitude and rate are radically different in air and inert media. The oxidizing atmosphere apparently accelerates the thermal degradation of the polymer, and/or supplements it with thermal oxidative processes. This leads to a loss of almost 100% of the polymer mass at ~730 °C. This process is accompanied by a sharp exothermic effect observed in the temperature range of 550–750 °C on the DSC thermogram (Figure 6b). At the same time, heating above 550 °C in an inert atmosphere leads to a gradual loss of another 25% in weight up to 1000 °C, without any exothermic effects. For clarity, Figure 6c shows the derivative of the thermogravimetric curve (DTG), which reflects the maximum weight loss of the sample as a function of temperature, as well as a comparison of the intensity of weight loss in inert and air media.

A similar thermal behavior was described for similar fully aromatic polyester containing 3,4-biphenylene unit based on 3,4-bibenzoic acid and hydroquinone [16]: these copolymers show analogous values of weight loss up to 500 °C and *T*_Δ5%_. The values of the glass points for those copolymers with a high content of bibenzoate are also about 180–190 °C.

**Figure 7:**
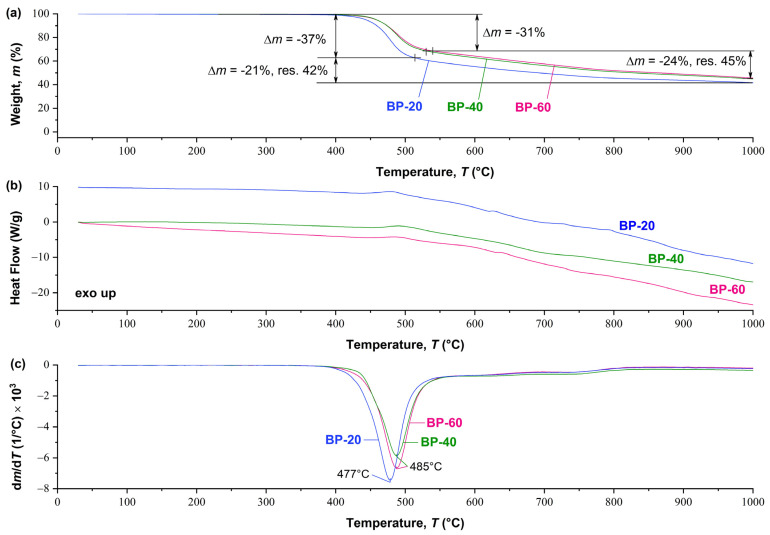
Comparison results of (**a**) TGA, (**b**) DSC, and (**c**) DTG analysis for BP-20, BP-40, and BP-60 copolyesters obtained in an inert (argon) atmosphere.

The thermal behavior of copolyesters with different content of 3HBCA units is almost identical (Figure 7). At the same time, it should be noted that with an increase in the ratio of 3HBCA:3HBA, there is a slight increase in the thermal stability of copolyesters: the peak of maximum weight loss shifts by about +10 °C and the intensity of thermal degradation also decreases. Similar observations were previously described for ternary copolymers 4HBA-4HBCA-3HBA [26]: the addition of a “kinked” comonomer (3HBA) led to the disappearance of thermal effects on DSC curves, as well as to a slight decrease in the thermal stability of the copolyesters. Obviously, in 3HBCA-3HBA copolyesters, a slight decrease in thermal stability upon the introduction of 3HBA is leveled by a significantly higher content of the 3HBCA biphenyl comonomer with higher thermal stability confirmed by the DSC-TGA results.

Thus, the significantly increased glass transition temperatures in combination with a sufficiently high temperature *T*_Δ5%_ allow us to state that the 3HBCA-3HBA copolyesters have a higher thermal stability not only in comparison with the semi-aromatic polyesters PET-4HBCA [21], but also in comparison with the aromatic copolyesters 4HBCA-4HBA-3HBA [26] obtained earlier.

### 3.3. Rheological Characteristics

A change in the composition of copolymers naturally affects the rheological characteristics of their melts. As previously described, the introduction of up to 80% of biphenyl units of 4HBCA into the main chain of PET leads to multiple increases in all viscoelastic properties of the copolymers [21].

**Figure 8:**
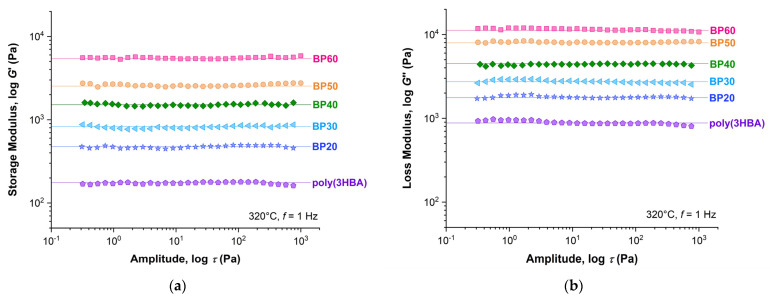
Dependences of the storage (**a**) and loss (**b**) moduli on the stress amplitude for homopolymer poly(3HBA) and BP-20–BP-60 copolyester melts at 320 °C.

Figure 8 shows the dependence of the storage (*G*′) and loss (*G*″) moduli on the stress amplitude in an oscillation regime with a frequency of 1 Hz for poly(3HBA) and BP-20–BP-60 melt at 320 °C. With an increase in 3HBCA content from 20 to 60%, a significant increase in viscous and elastic properties of the melts was observed. The difference between the *G*′ and *G*″ values decreased in order from BP-20 to BP-60, but the viscous characteristics prevailed over elastic ones for all (co)polymers in the entire deformation range. In addition, all copolyesters exhibited a very wide range of linear viscoelasticity (LVE)—about four orders of magnitude in stress amplitude values.

**Figure 9:**
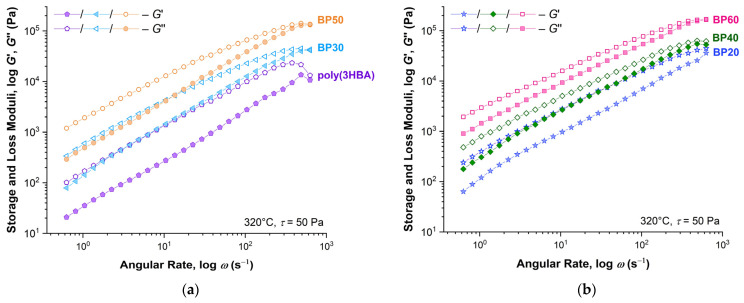
Dependences of the storage and loss moduli on angular rate with an amplitude of 50 Pa for homopolymer poly(3HBA) and BP-20–BP-60 copolyester melts at 320 °C.

The stress value of 50 Pa was used to determine the frequency sweep of the *G*′ and *G*″ moduli at 320 °C, shown in Figure 9a for samples poly(3HBA), BP30, and BP50 and in Figure 9b for samples BP20, BP40, and BP60 (division into two figures to prevent the intersection of data series). For all samples, the values of *G*″ exceeded the values of *G*′ in almost the entire frequency range, which indicates a significant prevalence of the viscous characteristics over the elastic ones in polyester melts. With an increase in the oscillation frequency to 100 Hz, the values of the moduli became equal. In addition, the difference between the *G*′/*G*″ values decreased with an increase in the content of rigid biphenyl units of 3HBCA.

**Figure 10:**
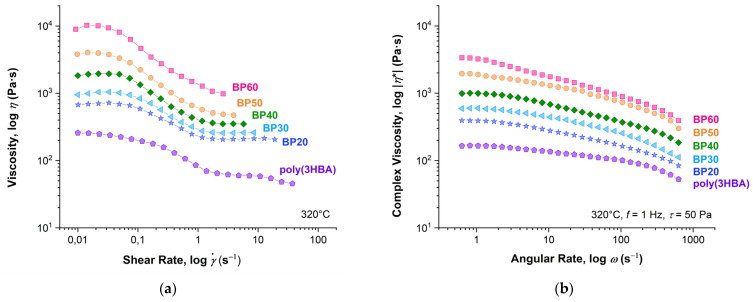
Flow curves of homopolymer poly(3HBA) and BP-20–BP-60 copolyester melts obtained under (**a**) stationary shear and (**b**) oscillation regimes at 320 °C.

Figure 10 presents flow curves obtained in the stationary shear regime (0.01–100 s^–1^) and in the oscillation mode (angular rate range 0.5–1000 s^–1^) for (co)polyester melts. All samples exhibit non-Newtonian behavior. With an increase in the 3HBCA content, the viscosity values increased drastically and shear thinning became more prominent. The complex viscosity values (|η*|) obtained by oscillation tests were at least twice as high as the shear viscosity values (*η*) at the condition of equality of shear and angular rates. This means that the Cox–Merz rule [27] does not work for these polymer melts. This can be explained by the orientational alignment in the flow, which proceeds more efficiently in shear flow for macromolecules of rigid-chain polymers. It is also worth noting that shear viscosity can only be measured up to shear rates of about 5–10 s^–1^ due to the high elasticity of 3HBA:3HBCA copolyester melts. After that, the spurt effect is observed. It should also be noted that melt viscosities are more likely to correspond to the molecular weights of the copolyesters determined by NMR rather than GPC.

**Figure 11:**
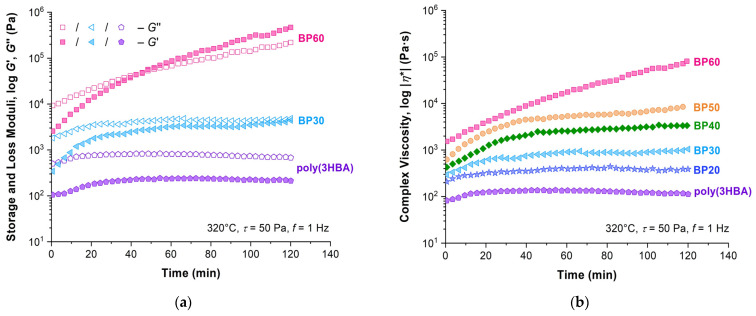
Evolution of the (**a**) complex modulus components and (**b**) complex viscosity values in time at strain of 50 Pa and frequency of 1 Hz for melts indicated in plots at 320 °C.

The significant increase in viscoelastic characteristics after exposure in time under a load was observed for all samples (Figure 11). The rate of their growth and the position of the crossover point of *G*′/*G*″ depended on the composition being inversely proportional to the 3HBCA content. Thus, increases in moduli and complex viscosity are not significant for the poly(3HBA) homopolymer, and the intersection of the *G*′/*G*″ curves did not occur. With an increase in 3HBCA content, the differences between the viscosity values measured at zero-time and after 2 h increased significantly. At the same time, the crossover point was reached after an exposure of 2 h for BP30 and 50 min for BP60.

An increase in the viscoelastic characteristics in time under a constant load is not unusual for copolyesters of such structures. Similar behavior was found earlier for copolyesters of PET-4HBA [28] and PET-4HBCA [21]. It was shown that the elastic characteristics increased faster than the viscous ones over time, leading to the appearance of the crossover point, after which *G*′ > *G*″. It is remarkable that the effect of an increase in the viscoelastic characteristics in time was also observed in an inert atmosphere, which eliminated the influence of thermal oxidative processes. Moreover, the crossover transition occurred later for copolymers with a high content of mesogenic units. The observed increase in moduli may be caused by a chemical reaction in macromolecules of copolyesters of PET and 4HBA or 4HBCA caused by their presence in diethylene glycol (DEG) units [21,28]. The reduced amount of DEG units in the copolyester backbone with a high content of 4HBA/4HBCA causes a longer period for reaching the crossover point.

The previous explanation for modulus evolution due to degradation caused by DEG units is not suitable for fully aromatic 3HBCA-3HBA copolymers. In addition, DSC/TGA data obtained both in an inert atmosphere and in air (Figure 6) did not demonstrate any change in polymers up to 350 °C. Moreover, the increase in 3HBCA content resulted in a higher thermal stability. A possible explanation for this consists of the reorientation of macromolecules under prolonged oscillatory loading, which occurs in melts of 3HBCA-3HBA rigid-chain copolyesters and leads to the strengthening of the reorganized internal structure. The lesser the mobility of macromolecules, i.e., with an increase in the content of 3HBCA, the longer the process. This hypothesis is supported by the fact that for less rigid-chain poly(3HBA) and for copolyesters with a low content of 3HBCA biphenyl units, the transition through the crossover was not observed or occurred noticeably later in comparison with the BP60 sample.

### 3.4. Polarization Optical Microscopy

**Figure 12:**
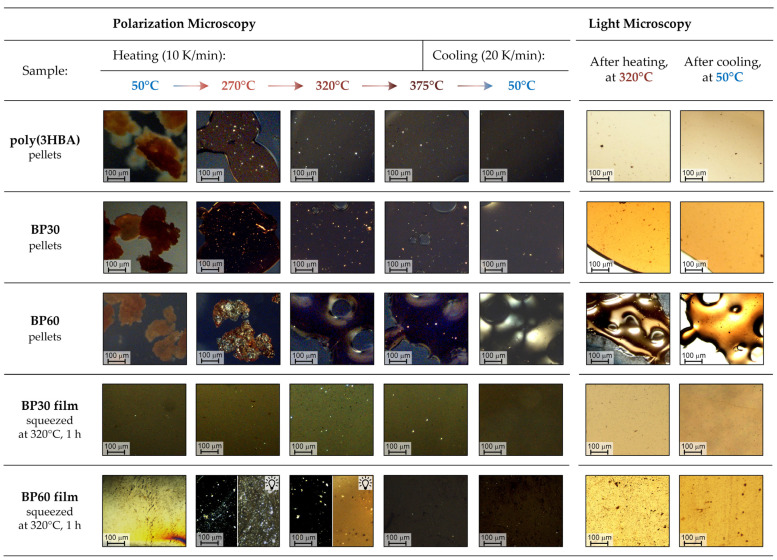
Micrographs of poly(3HBA) and BP30/60 melts obtained with and without crossed polarizers during heating to 375 °C and subsequent cooling to 50 °C.

In order to observe optical properties of synthesized copolyesters, their melts were analyzed by polarizing microscopy. Polymer samples (pellets), placed between two glass slides, were sequentially heated up to 375 °C (with image capture at 50, 150, 270, 320, and 375 °C) and then cooled up to 50 °C. The micrographs obtained with/without using the crossed polarizers are shown in Figure 12.

As can be seen from the micrographs, all (co)polyesters softened after the glass transition temperature (above 150–190 °C); at 270 °C, a rather high plasticity of the melts is observed. Upon further heating and subsequent cooling, the homopolymer poly(3HBA), as well as the copolyester with a low content of biphenyl units (BP30), form homogeneous, optically transparent films. At a high content of 3HBCA fragments (sample BP60), the viscosity of the melt becomes excessively high even at 375 °C, which prevents the formation of a homogeneous film between glass slides.

For a more accurate evaluation of the melt isotropy for samples BP30 and BP60, thin films were prepared by pressing polymer powder under a load of ~5 kg at 320 °C for 1 h. Further, these films were analyzed via the same protocol (micrographs are also shown in Figure 12). It can be noted that for BP60 at 270–320 °C, the presence of micron-sized particles with birefringence is observed in the melt. It is most probable that these particles are high-melting partially crystalline regions rather than LC phase domains. It is possible that the reason for the strong growth of modules during the long-term exposure of the BP60 melt at the temperature of the existence of these particles (Figure 11a above) is some kind of evolution of these crystalline regions under conditions of external mechanical impact (e.g., recrystallization, growth). With further heating, these regions melt, and the sample becomes almost isotropic. After cooling, the melt forms an optically transparent glassy film with high hardness.

The obtained samples did not exhibit the optical properties inherent for nematic melts of thermotropic polyesters, as was previously shown for polymers based on 3,4BDCA-HQ (HQ—hydroquinone derivatives) [16] and 4HBA-4HBCA-3HBA [26]. However, in the works listed above, bent *m*-substituted comonomers (3,4BDCA and 3HBA) were part of the composition in addition to *p*-substituted mesogenic units. In these cases, the introduction of *m*-substituted fragments expectedly reduced the crystallinity of the copolyesters, but did not prevent their transition to the nematic LC state.

In our case, the possibility that the spontaneous formation of the LC phase is likely to be suppressed for (co)polyesters was entirely formed from *m*-substituted rigid fragments (3HBCA and 3HBA) and block sequences were absent. Thus, it was confirmed that 3HBCA is apparently not a mesogenic monomer and neither is 3HBA.

## 4. Conclusions

A series of new wholly aromatic (co)polyesters based on *m*-substituted bifunctional comonomers—4′-hydroxybiphenyl-3-carboxylic (3HBCA) and 3-hydroxybenzoic (3HBA) acids with molar ratios of 3HBCA:3HBA from 0:100 to 60:40) was synthesized.

NMR and FTIR spectroscopy methods proved the full compliance of the copolymer compositions with the target ratios of comonomers, as well as high compositional homogeneity (absence of block sequences). The *M*_n_ values determined by NMR were approx. 10,000–20,000 g/mol. Slightly overestimated values were obtained by the GPC method: *M*_n_~15,000–25,000 and *M*_w_~50,000–100,000 g/mol. The intrinsic viscosity of the obtained copolyesters was in the range of 0.6–0.8 dL/g.

Thermal analysis showed that all 3HBCA-3HBA copolyesters are amorphous, and with an increase in the content of biphenyl units (3HBCA), the glass transition temperature increased significantly: from 146 °C (poly3HBA) to 186 °C (60 mol.% of 3HBCA). According to the TGA data, the onset of intense thermal decomposition of the synthesized polyesters occurred above 450 °C. Thus, this indicates a sufficiently high thermal stability of these polyesters.

Rheological studies have shown that copolyester melts exhibit non-Newtonian behavior. In the case of copolyesters with a high content of 3HBCA, under prolonged non-destructive deformation (oscillation regime), increases in ordering could be observed as a result of the transition of the melt through the crossover point from a predominantly viscous state to a predominantly elastic one.

At the same time, the method of polarization optical microscopy did not confirm the possibility of the existence and the conditions for the transition to the liquid crystal state for these polyesters. For (co)polyesters entirely formed from *m*-substituted rigid fragments (3HBCA and 3HBA), in combination with the absence of block sequences, the possibility of spontaneous formation of the LC phase is probably blocked. These results confirm that it is possible to obtain high-performance copolyesters based on 3HBCA, but that it is not a mesogenic comonomer. This fact can be used to control the anisotropic properties of synthesized copolyesters in the future.

## Figures and Tables

**Table 1:** Composition, intrinsic viscosity ([*η*]), molecular weight (*M*_n_, *M*_w_), and polydispersity index (*PDI*) of 3HBCA-3HBA polyesters defined by ^1^H NMR (in a mixture of CDCl_3_:CF_3_COOH or CD_3_OD/D_2_O/NaOD) and GPC methods.

Sample	Targeted 3HBCA:3HBA mol Ratio	Founded 3HBCA:3HBA mol Ratio ^a^	Founded 3HBCA:3HBA mol ratio ^b^	[*η*] dl/g	*M*_n_ ^a^ kg/mol	*M*_n_ ^b^ kg/mol	*M*_n_ ^c^ kg/mol	*M*_w_ ^c^ kg/mol	*PDI* ^c^
BP60	60:40	62:38	61:39	0.48	18.6	18.6	*insoluble*		
BP50	50:50		51:49	0.80		10.7	*insoluble*		
BP40	40:60	42:58	42:58	0.66	7.7	10.2	18.6	48.0	2.6
BP30	30:70		32:68	0.73		14.7	16.6	55.5	3.3
BP20	20:80		21:79	0.81		12.3	23.9	94.5	4.0
poly(3HBA)	0:100	0:100	0:100	0.58	11.8	11.3	15.8	100.4	6.3

^a^ Calculated from ^1^H NMR spectroscopy data (CD_3_OD/D_2_O/NaOD hydrolyzed solution). ^b^ Found by ^1^H NMR spectroscopy (CDCl_3_/CF_3_COOH solution). ^c^ Calculated from GPC results (eluent—CHCl_3_, PS standard).

**Table 2:** Dyad sequence distribution and sequence order parameter (*Ψ*_A_, *Ψ*_B_, *Ψ*_av._ = (*Ψ*_A_ + *Ψ*_B_)/2) for copolyesters BP50 and BP60 (A—3HBCA, B—3HBA) calculated from the intensity of the corresponding signals in ^13^C spectra.

Sample	Dyad Sequence Distribution, %						*Ψ*_A_	*Ψ_B_*	*Ψ_av._*
	AA	AB	AA + AB	BA	BB	BA + BB			
BP60	37.0	24.4	61.4	23.6	15.0	38.6	0.024	−0.005	0.009
BP50	26.9	24.7	51.6	24.2	24.2	48.4	−0.011	−0.032	−0.021

**Table 3:** Relative intensities of bands 744 and 804 corresponding to the 1,3- and 1,4-substituted benzoic ring (*ν*_CCH_) of copolyesters BP20, BP40, and BP60. Normalization was performed using the intensity of the carbonyl group at 1733 cm^−1^.

Sample	Value of Ratio I_744_/I_804_	Comonomer Ratio 3HBA:3HBCA Determined	
		by FTIR	by ^1^H NMR
BP20	4.7	82:18	79:21
BP40	2.7	73:27	58:42
BP60	1.9	35:65	39:61

## Data Availability

The data presented in this study are available upon request from the corresponding author.

## Supplementary Materials

The following supporting information can be downloaded at: https://www.mdpi.com/article/10.3390/polym15092133/s1, Figure S1: ^1^H spectrum of 3ABCA recorded in a mixture of CDCl_3_:CF_3_COOH; Figure S2: COSY 2D ^1^H-^1^H spectrum of 3ABCA recorded in a mixture of CDCl_3_:CF_3_COOH; Figure S3: HSQC 2D ^1^H-^13^C spectrum of 3ABCA recorded in a mixture of CDCl_3_:CF_3_COOH; Figure S4: COSY 2D ^1^H-^1^H spectrum of BP50 recorded in a mixture of CDCl_3_:CF_3_COOH; Figure S5: HSQC 2D ^1^H-^13^C spectrum of BP50 recorded in a mixture of CDCl_3_:CF_3_COOH; Figure S6: ^13^C spectrum of BP60 recorded in a mixture of CDCl_3_:CF_3_COOH; Table S1: Signals in ^13^C spectra of BP50 and BP60 copolyesters and their assignment.
