# Supplementary material for: Synthesis and Characterization of Novel Wholly Aromatic Copolyesters Based on 4′-Hydroxybiphenyl-3-Carboxylic and 3-Hydroxybenzoic Acids
Source: Polymers (Basel). 2023 Apr 29;15(9):2133. doi: 10.3390/polym15092133 (PMC10180978; doi:10.3390/polym15092133)
Supplement: Supplementary file 1 [file polymers-15-02133-s001.zip › polymers-2360206-supplementary.pdf]

polymers

Article

Synthesis and Characterization of Novel Wholly Aromatic Copolyesters Based on 4′-Hydroxybiphenyl-3-carboxylic and 3-Hydroxybenzoic Acids

Pavel A. Mikhailov *, Kirill V. Zuev * and Valery G. Kulichikhin

A. V. Topchiev Institute of Petrochemical Synthesis, Russian Academy of Sciences (TIPS RAS), 29 Leninsky Prospekt, 119991 Moscow, Russia; klch@ips.ac.ru
* Correspondence: pmih@ips.ac.ru (P.A.M.); zuev.kirill@inbox.ru (K.V.Z.)

Suplementary materials

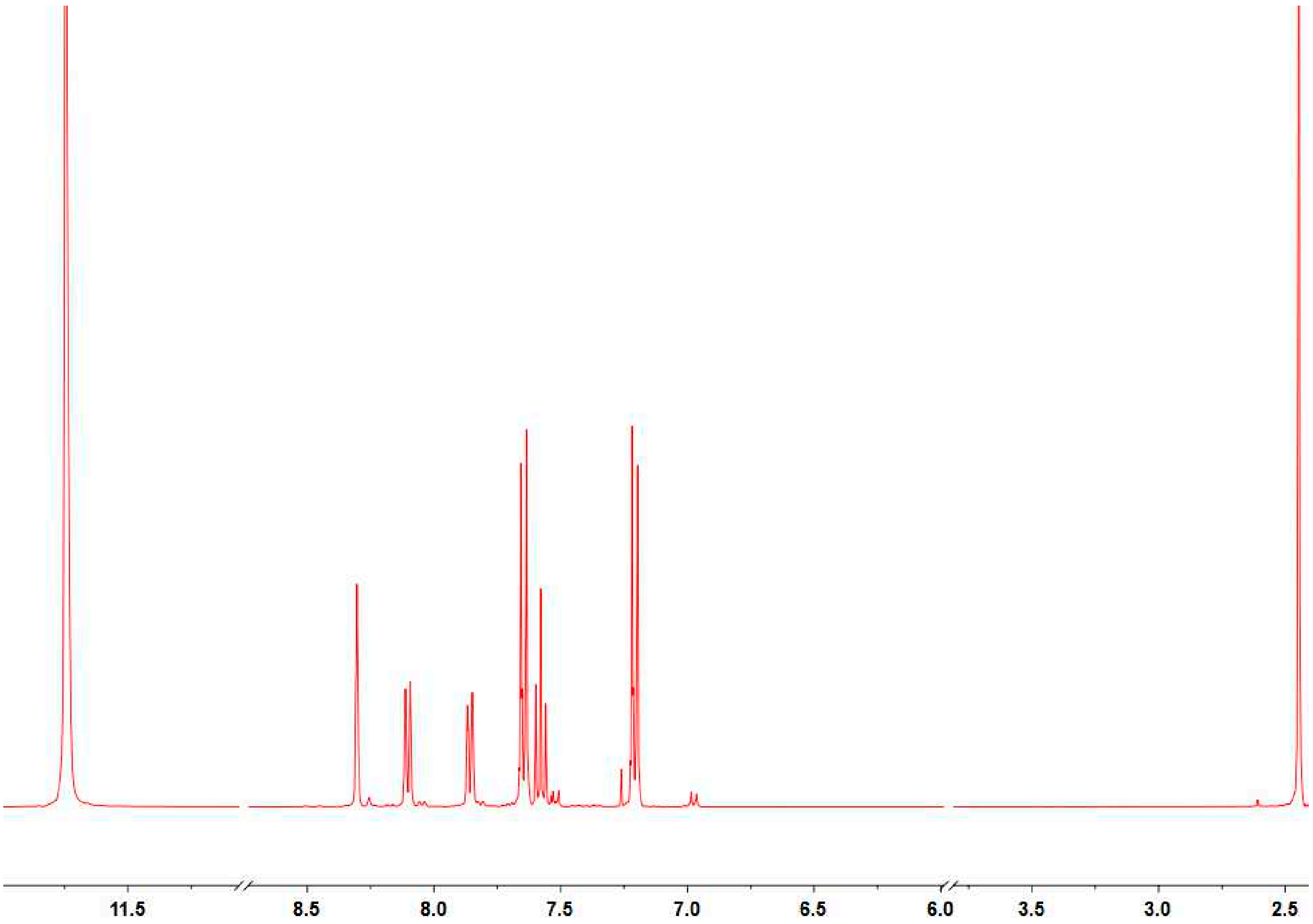

Figure S1. ^{1}H Spectrum of 3ABCA recorded in a mixture $CDCl_3$:CF_3COOH.

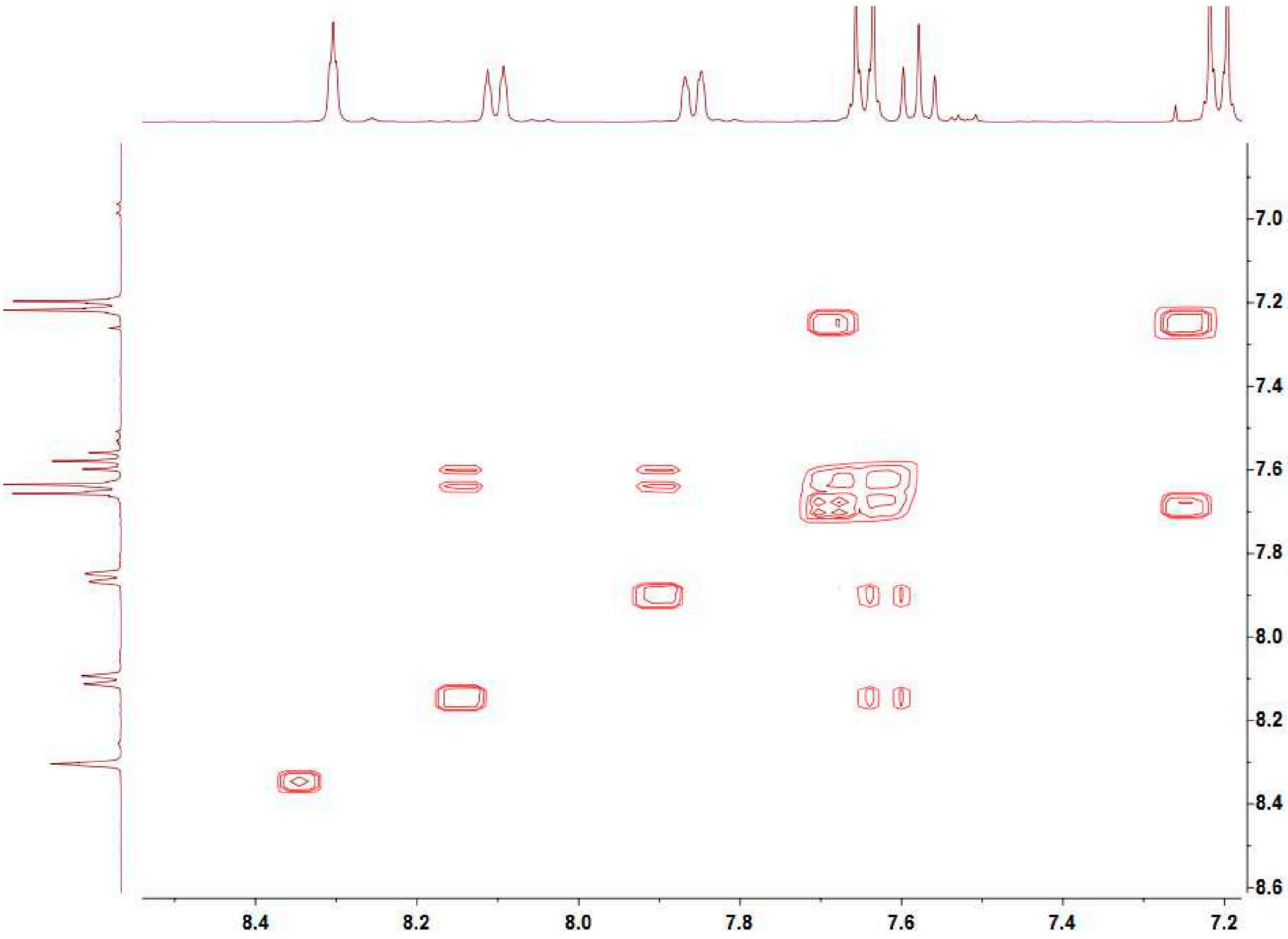

Figure S2. COSY 2D ^1H-^1H spectrum of 3ABCA recorded in a mixture $CDCl_3$:CF_3COOH.

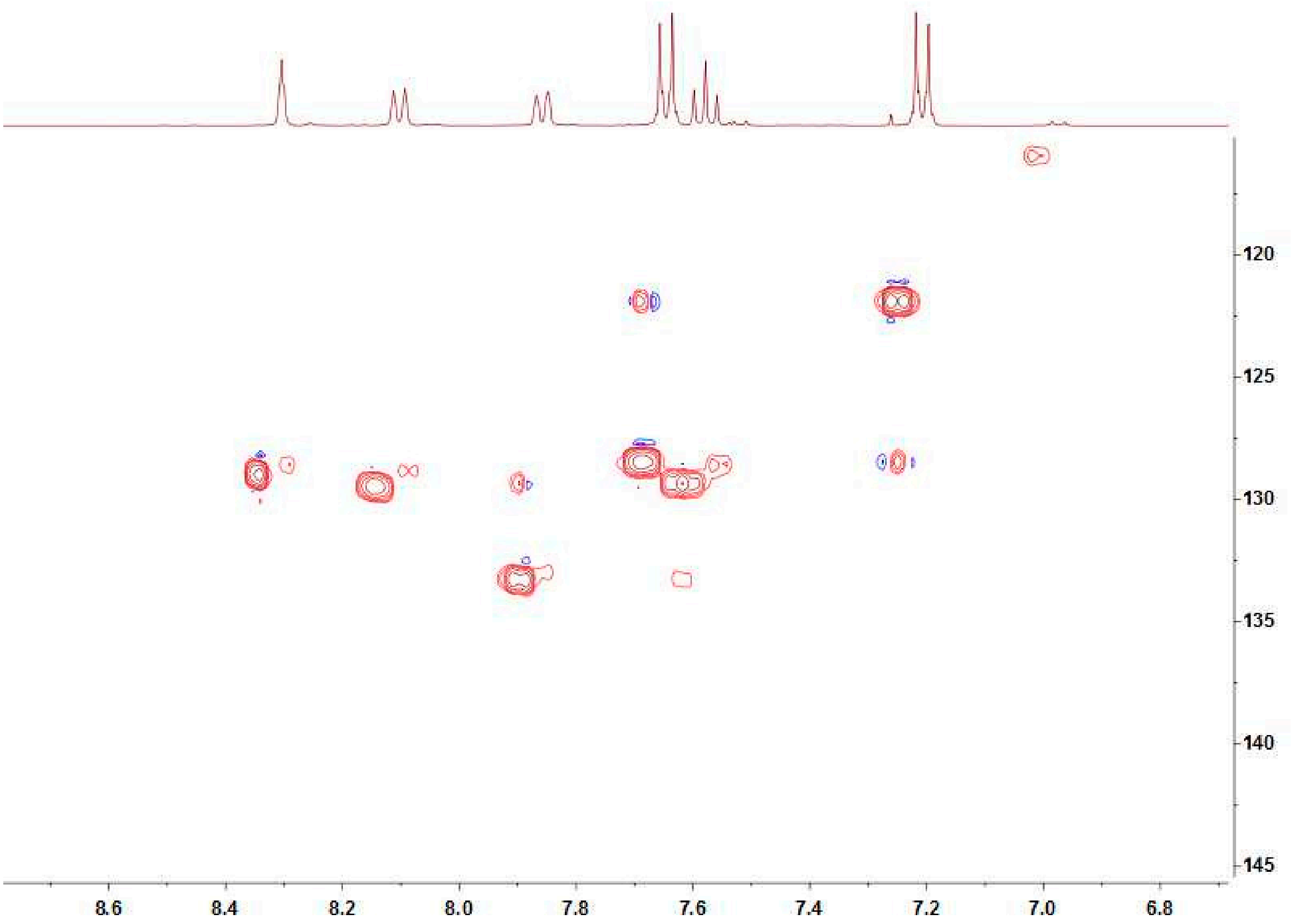

Figure S3. HSQC 2D ^1H-^{13}C spectrum of 3ABCA recorded in a mixture $CDCl_3$:CF_3COOH.

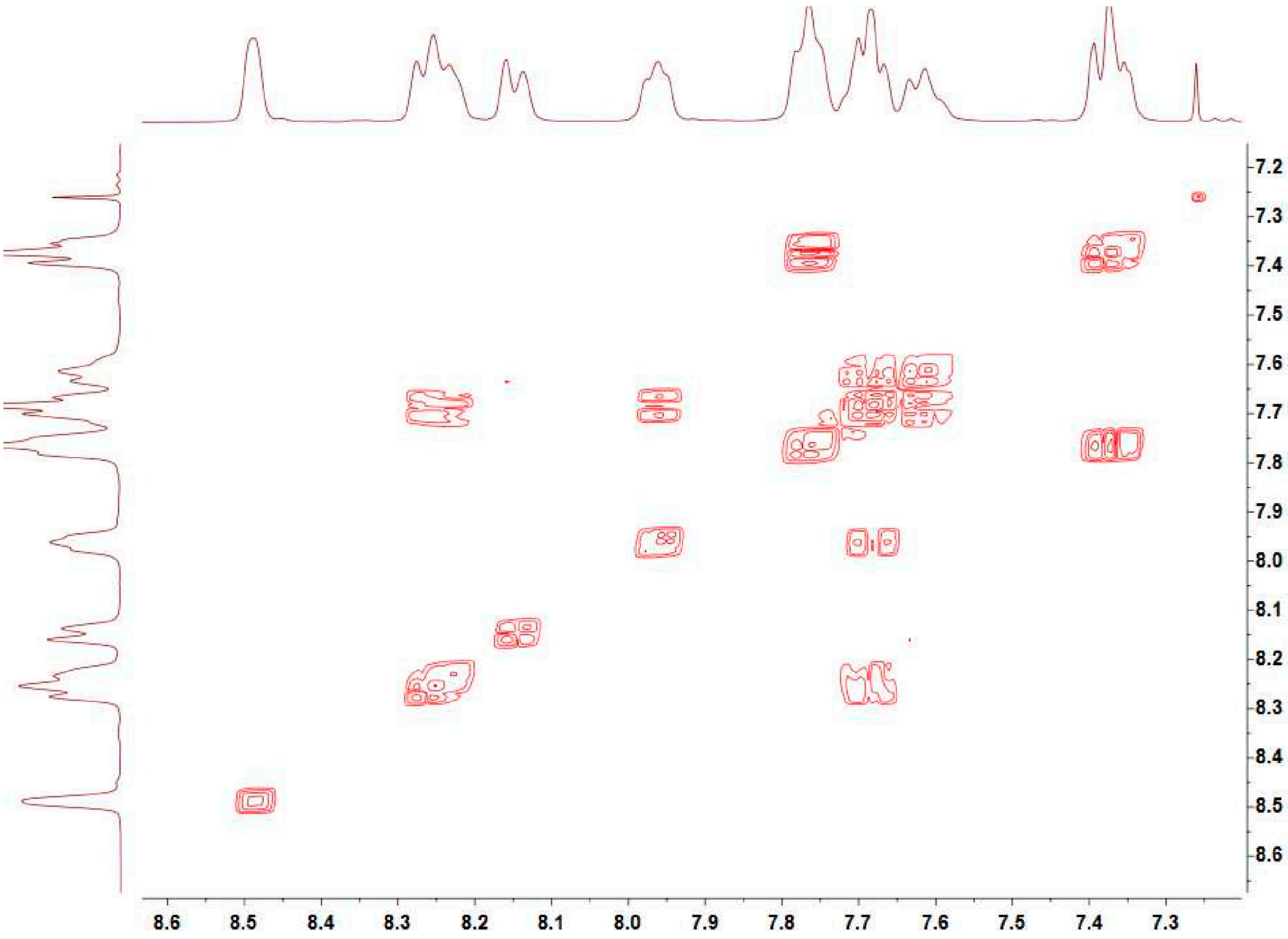

Figure S4. COSY 2D ^1H-^1H spectrum of BP50 recorded in a mixture $CDCl_3$:CF_3COOH.

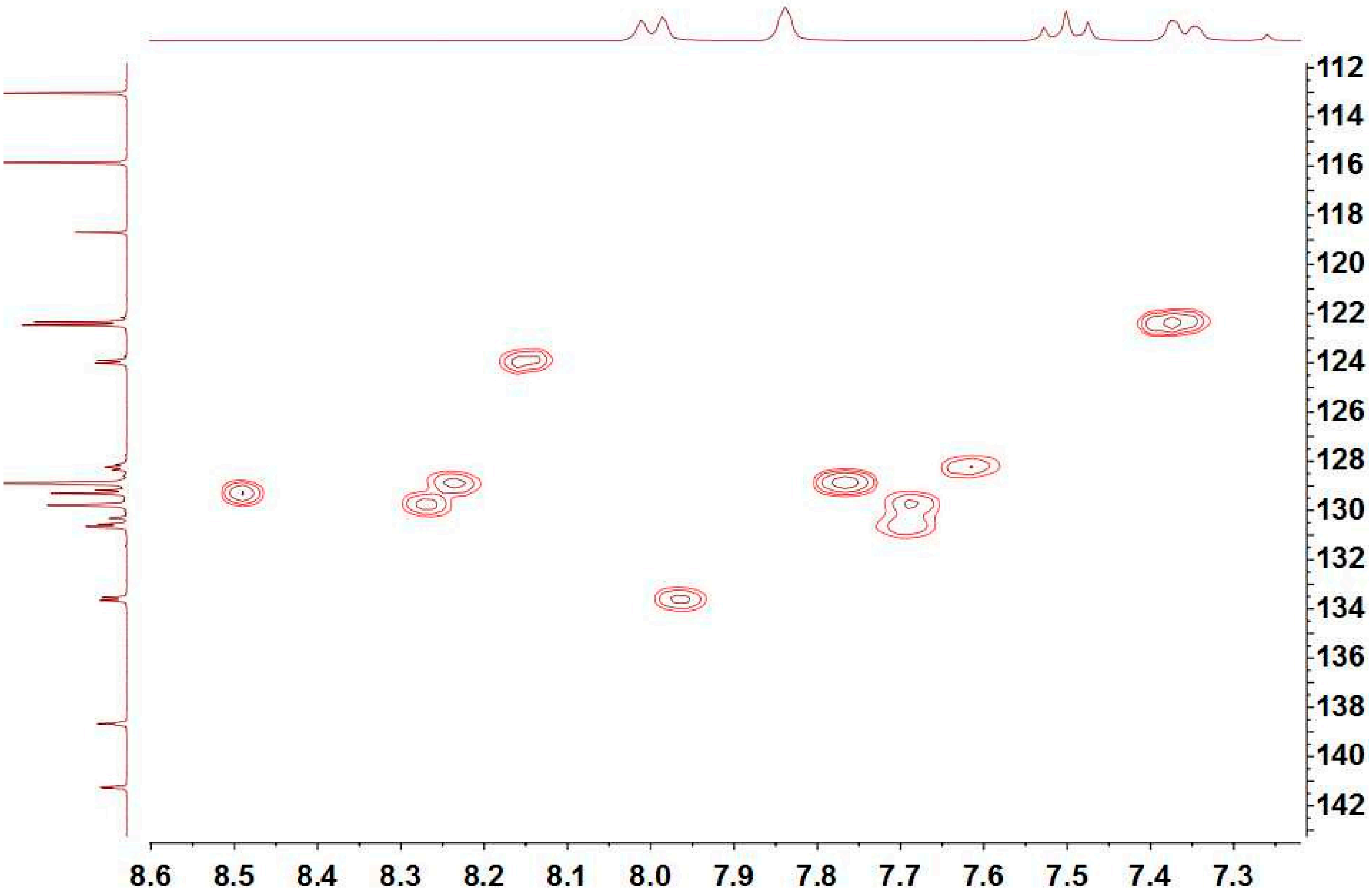

Figure S5. HSQC 2D ^1H-^{13}C spectrum of BP50 recorded in a mixture $CDCl_3$:CF_3COOH.

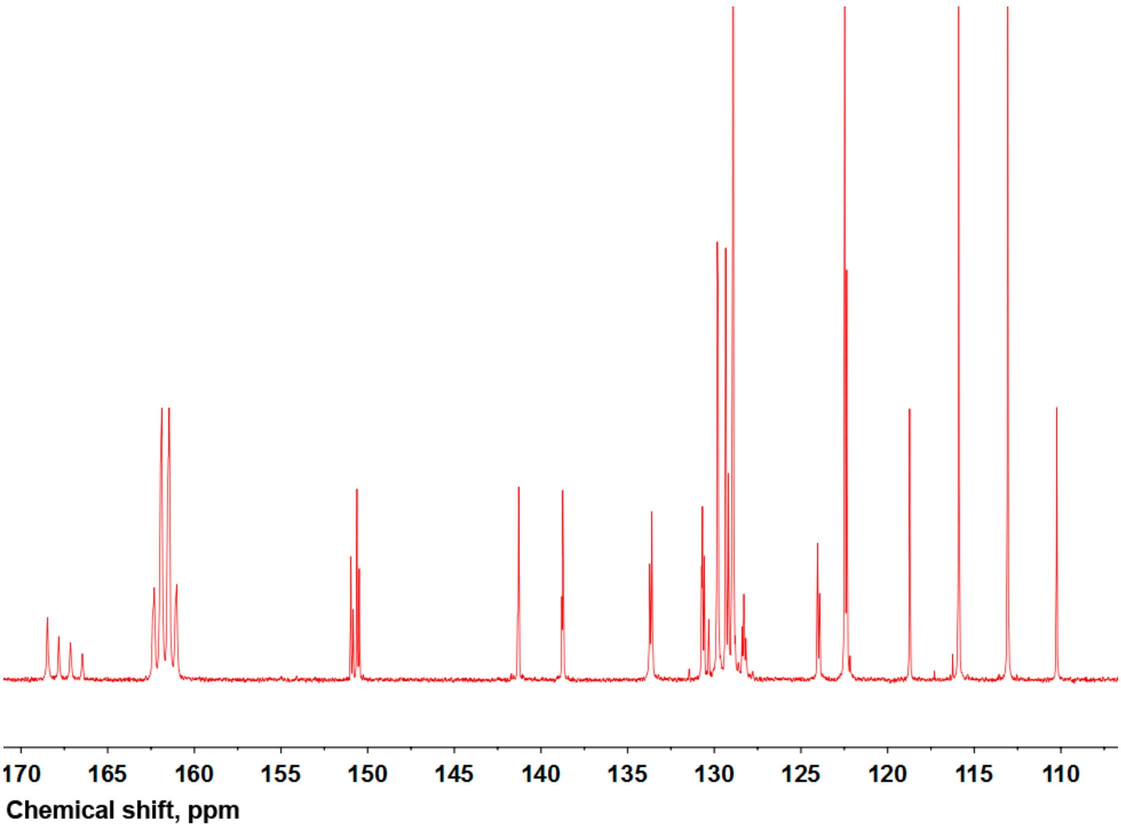

Figure S6. ^{13}C spectrum of BP60 recorded in a mixture $CDCl_3$:CF_3COOH.

Table S1. Signals in ^{13}C spectra of BP50 and BP60 copolyesters and their assignment.

BP50 Chemical shift	BP60 Chemical shift	Assignment	Position
168.31	168.42	4'3HBCA carbonyl	4'3HBCA-4'3HBCA dyad
167.65	167.82	4'3HBCA carbonyl	4'3HBCA-3HBA dyad
166.99	167.15	3HBA carbonyl	3HBA-4'3-HBCA dyad
166.30	166.46	3HBA carbonyl	3HBA-3HBA dyad
150.94	150.97	O-Ar (4'3HBCA - 4' and 3HBA -3)	
150.81	150.83		
150.60	150.62		
150.47	150.48		
141.29	141.34	Quarternary atoms	
141.24	141.28		
138.72	138.80		
138.67	138.74		
138.62	138.69		
133.66	133.72	4'3HBCA	4
133.54	133.60	4'3HBCA	4
130.68	130.73	3HBA	6
130.64	130.68	3HBA	6
130.56	130.58	3HBA	6
130.30	130.32	3HBA	6
129.80	129.83	4'3HBCA	2',6
129.76	129.79		
129.31	129.34	4'3HBCA	2
129.18	129.18	3HBA - 2; 4'3HBCA -2',6'	
128.93	128.92		
128.35	128.40	3HBA	5
128.24	128.29	3HBA	5
128.13	128.18	3HBA	5
124.02	124.04	3HBA	4
123.90	123.92	3HBA	4
122.47	122.48	4'3HBCA	3',5'
122.35	122.36	4'3HBCA	3',5'
118.70	118.72	Quarternary atoms	
115.87	115.90		
113.04	113.07		
110.21	110.24		
